# A versatile, automated and high-throughput drug screening platform for zebrafish embryos

**DOI:** 10.1242/bio.058513

**Published:** 2021-09-02

**Authors:** Alexandra Lubin, Jason Otterstrom, Yvette Hoade, Ivana Bjedov, Eleanor Stead, Matthew Whelan, Gaia Gestri, Yael Paran, Elspeth Payne

**Affiliations:** 1 Research Department of Haematology, Cancer Institute, University College London, London WC1E 6DD, UK; 2 IDEA Bio-Medical Ltd., Rehovot 76705, Israel; 3 Research Department of Cancer Biology, Cancer Institute, University College London, London WC1E 6DD, UK; 4 Division of Infection and Immunity, University College London, London WC1E 6BT, UK; 5 Department of Cell and Developmental Biology, University College London, London WC1E 6AR, UK

**Keywords:** Drug screening, High-throughput, Zebrafish

## Abstract

Zebrafish provide a unique opportunity for drug screening in living animals, with the fast-developing, transparent embryos allowing for relatively high-throughput, microscopy-based screens. However, the limited availability of rapid, flexible imaging and analysis platforms has limited the use of zebrafish in drug screens. We have developed an easy-to-use, customisable automated screening procedure suitable for high-throughput phenotype-based screens of live zebrafish. We utilised the WiScan^®^ Hermes High Content Imaging System to rapidly acquire brightfield and fluorescent images of embryos, and the WiSoft^®^ Athena Zebrafish Application for analysis, which harnesses an Artificial Intelligence-driven algorithm to automatically detect fish in brightfield images, identify anatomical structures, partition the animal into regions and exclusively select the desired side-oriented fish. Our initial validation combined structural analysis with fluorescence images to enumerate GFP-tagged haematopoietic stem and progenitor cells in the tails of embryos, which correlated with manual counts. We further validated this system to assess the effects of genetic mutations and X-ray irradiation in high content using a wide range of assays. Further, we performed simultaneous analysis of multiple cell types using dual fluorophores in high throughput. In summary, we demonstrate a broadly applicable and rapidly customisable platform for high-content screening in zebrafish.

This article has an associated First Person interview with the first author of the paper.

## Abstract (precis)

**Summary:** We developed and validated a fast, flexible and rapidly customisable platform, automating both image acquisition and quantitative analysis, for high-content drug screening in live zebrafish embryos.

## INTRODUCTION

Zebrafish provide an excellent model for human disease and offer a unique opportunity for *in vivo* small-molecule phenotypic drug screening. Each breeding pair can lay hundreds of embryos, which, combined with the rapid development and transparent nature of the embryos, makes them amenable to microscopy-based screens usually otherwise restricted to cell culture. Unlike most *in vivo* screening platforms, thousands of animals can be imaged within days, allowing for a relatively high-throughput screen, with the advantages of screening in intact living animals.

The utility of such *in vivo* screens has been demonstrated by the rapid repurposing of identified compounds into clinical trials. This is exemplified by dmPGE2, which entered clinical trials as a therapy for patients undergoing umbilical blood cord transplantation, having been found to enhance haematopoietic stem cells in a zebrafish screen using *in situ* hybridisation (North et al., 2007; Hagedorn et al., 2014). Additionally, ORC-13661, identified in zebrafish screens of hair cells in zebrafish embryos (Owens et al., 2008; Chowdhury et al., 2018; Kitcher et al., 2019) is currently in clinical trials as an agent to prevent hearing loss from aminoglycoside antibiotic-induced hair loss.

Despite their advantages, the limited availability of image acquisition and, especially, analysis platforms supporting zebrafish in a fast, flexible format has limited their widespread uptake in drug screens. Screens are typically slow, often relying on manual or bespoke imaging solutions and manual analysis. For example, North et al. (2007) utilised a manual qualitative scoring after *in situ* hybridisation using two probes, to identify dmPGE2. The discovery of ORC-13661 as a modifier of hair cells also relied on manual inspection of fluorescent neuromasts for 10,960 compounds after treatment with a dye, with manual counting required to quantify changes (Owens et al., 2008). This hair cell assay is rapid and simple and has been used in a number of additional screens (Chiu et al., 2008; Vlasits et al., 2012; Esterberg et al., 2013; Pei et al., 2018); however, all still rely on manual counting or scoring of individual fish, creating a significant bottleneck in analysis throughput.

Zebrafish have also emerged as a valuable patient-derived xenograft model for drug screening for novel chemotherapeutic compounds or to identify patient-specific responses, having several advantages over mouse models including far more rapid time scales, cost effectiveness and the ability to use large numbers (Xiao et al., 2020; Fazio and Zon, 2017; Fior et al., 2017; Jung et al., 2012; Tonon et al., 2016). Zebrafish xenografts have been used successfully to identify or validate chemotherapeutic compounds, for example to validate BPIQ against lung cancer cells (Chiu et al., 2015) and the identification of regorafenib against adenoid cystic carcinoma (Chen et al., 2017). Although there have been a number of examples of how zebrafish could provide a useful high-throughput screening platform for xenograft models (Haney et al., 2020; Zhang et al., 2014; Lin et al., 2019; Fu et al., 2018; Somasagara et al., 2021), the use of these on large scale has so far been limited. A platform with greater flexibility and ease of customisation would greatly aid the use of xenograft models for drug screening, owing to the variation and complexity between different models, and different read-outs for results, for example tumour size (Zhang et al., 2014) or cell count (Somasagara et al., 2021).

The optimal zebrafish high-throughput screening platform would permit automation of both image acquisition and analysis across a range of multiplexed assays and phenotypes with minimal human intervention. One of the primary challenges in this process is getting the embryo in the desired orientation for imaging without manual manipulation of the embryos. One approach has been to use small glass capillaries for imaging embryos, as in the VAST Bioimager, which allows for automated imaging of zebrafish embryos in a chosen orientation (Pulak, 2016; Pardo-Martin et al., 2010; Pardo-Martin et al., 2013; Chang et al., 2012; Haney et al., 2020). Zebrafish embryos can also be imaged in a semi-automated format by standard microscopes in 96-well plates (Romano and Gorelick, 2014), although there is limited control over the orientation of the fish without manual manipulation or inspection of the images.

Existing automated image analysis solutions are much more limited, with most platforms developed as bespoke solutions. One popular technique is to design algorithms to automatically analyse fluorescent images of transgenic zebrafish, or fish fluorescently labelled with a dye. A number of such screens have adapted the ImageXpress High Content Screening System by Molecular Devices for automated image acquisition. This has included assays to quantify the number of angiogenic blood vessels (Tran et al., 2007), to analyse axon length (Kanungo et al., 2011) and to measure tumour size (Zhang et al., 2014) or count cells (Somasagara et al., 2021) in xenografted zebrafish. However, the automated analysis for these has often required either the development of bespoke algorithms, or the custom adaption of more general software. There are a number of open-source approaches that have been designed or adapted for analysis of zebrafish embryos. ImageJ is commonly used, for example to quantify fluorescence in xenografted zebrafish to assess chemotherapeutic compounds (Haney et al., 2020), although this relies on thresholding out autofluorescence, and can only be used to look at either the whole fish or a manually selected region. QuantiFish is an open-source application developed for analysing fluorescent foci in zebrafish, which has been used to analyse bacterial infection in embryos (Stirling et al., 2020). CellProfiler is a more general image analysis platform (Kamentsky et al., 2011), which has a number of published pipelines, including for the analysis of zebrafish embryos to quantify haemoglobin (Metelo et al., 2015). However, to adapt these platforms for new applications requires a lot of development by the user to set up the analysis, which can be challenging without expertise, limiting widespread usage. Artificial Intelligence (AI)-based approaches can provide the opportunity for automated image analysis with broader applicability. Using Definiens Cognition Network Technology (CNT), Vogt et al. designed and trained an algorithm to detect and segment transgenic fluorescent embryos arrayed in 96-well plates and quantify blood vessel development (Vogt et al., 2009). They were then able to adapt this method to a different transgenic fish line and phenotype, which was used in a chemical screen for FGF signalling (Vogt et al., 2010; Saydmohammed et al., 2011).

We sought a screening platform to conduct small-molecule and genetic screens looking at haematopoietic stem and progenitor cells (HSPCs) in zebrafish embryos. We set out to develop an automated screen, with a long-term goal of screening for targeted therapeutics for myeloid malignancies. The *Tg(itga2b:GFP)* transgenic line, which labels thrombocytes and HSPCs with green fluorescence (Lin et al., 2005), provides a readout of the number and location of HSPCs. A semi-automated screen for HSPCs has previously been developed (Arulmozhivarman et al., 2016), but this screen required a custom analysis platform and user input to define the region of interest.

We have developed an easy-to-use, yet broadly applicable zebrafish embryo screening platform automating both image acquisition and quantitative analysis. We initially performed phenotypic validation for our screen of interest defining HSPCs using genetic and radiation screens of known phenotype. We then extended our assays to combine with multiple fluorescent outputs using mCherry-tagged myeloid cells, and further expanded our screen readout to include the apoptotic Acridine Orange for use in toxicity screens. To highlight the breadth and simplicity of the platform, we also utilised a hair cell marker that has previously been used in a number of manual screens and a brightfield morphological screen of eye size. Our customisable screen can easily and rapidly be applied to study of other anatomical sites and phenotypes for generalized drug screening in zebrafish, with automated image acquisition and analysis of thousands of fish in a single day.

## RESULTS

### Automatic detection of zebrafish embryos and internal anatomy in multiplexed fluorescence imaging with the co-developed WiSoft^®^ Athena image analysis platform

**Fig. 1.**
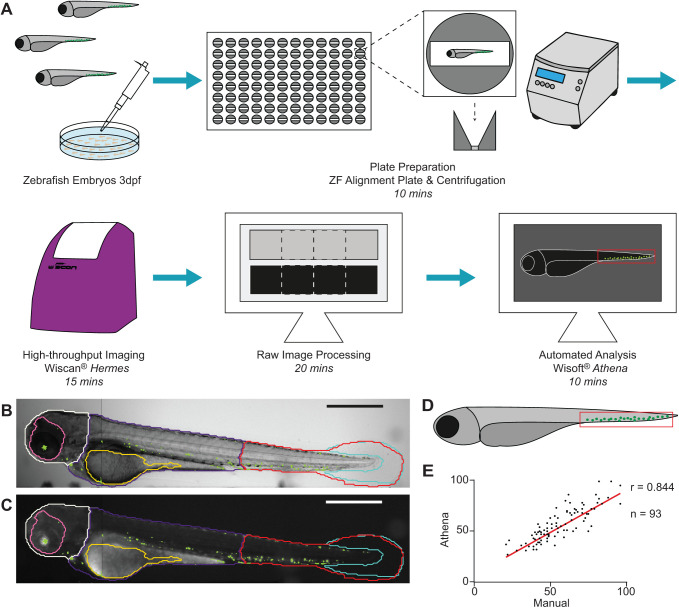
**The screening workflow and automated image analysis to detect zebrafish embryos, and count haematopoetic stem cells in the tails.** (A) Schematic of screening platform workflow including plate preparation, image acquisition and image analysis of zebrafish embryos with approximate timings. (B,C) Brightfield image (B) and fluorescent image (C) of a *Tg(itga2b:GFP)* zebrafish at 3 dpf acquired from the Hermes showing some of the regions identified by Athena: tail (red), trunk (dark blue), head (white), eye (pink), yolk (yellow), tail fin (light blue) and fluorescent granules (green). (D) Cartoon showing the location of haematopoietic stem and progenitor cells (HSPCs) in the caudal haematopoietic tissue (CHT) in the tail at 3 dpf. (E) Correlation of HSPC counts (between manual and granule) in 93 individual images of *Tg(itga2b:GFP)* embryos at 3 dpf using the Athena Zebrafish Application, analysed using simple linear regression with r=0.844. Scale bars: 500 µm. *n* refers to the number of embryos analysed.

Effective high-content screening (HCS) necessitates simple and fast image acquisition to allow for high throughput. We utilised the WiScan^®^ Hermes High Content Imaging System (IDEA Bio-Medical) to rapidly acquire both fluorescent and brightfield images of live zebrafish embryos at 3 days post-fertilisation (dpf). The workflow is depicted in Fig. 1A. Live phenylthiourea-treated embryos were anaesthetised and loaded into a 96-well zebrafish alignment plate (Hashimoto) with one embryo per well, using a manual pipette. The plate was briefly centrifuged, before imaging with the Hermes (Fig. 1A). Embryos were imaged using a 4× objective and *z*-stack acquisition of five slices, spanning 0.2 mm, with four overlapping images along each well to permit accurate image stitching and full-fish visualisation (Fig. S1). Image acquisition was carried out in brightfield and fluorescent channels, taking ∼15 min to obtain the 3840 raw images per full plate, allowing for the relatively high throughput. Image pre-processing steps are carried out automatically by the accompanying batch image processing software package (IDEA Bio-Medical, see Materials and Methods) in ∼20 min. We performed best *z*-slice selection (most in-focus *z*-plane) for the brightfield channel and maximum *z*-axis intensity projection for fluorescence channels prior to image stitching for full-fish analysis (Fig. S1), with images processed in batches by accompanying software. Fluorescence maximum intensity was chosen over best-slice selection to capture all fluorescently labelled cells throughout the fish volume. In this fashion, we were able to visualise individual fluorescently labelled cells without the need for additional processing or image deconvolution, while also having both the tail and head of the fish in proper focus for analysis of the brightfield channel.

IDEA Bio-Medical developed a novel zebrafish image analysis application for their WiSoft^®^ Athena software package that identifies the fish and internal anatomy with no required user input. This analysis application uses a unique deep-learning AI algorithm to analyse the brightfield images of full zebrafish (see Materials and Methods), trained utilizing hundreds of zebrafish images as input, in conjunction with multiplexed fluorescence channel analysis. Thirteen separate datasets were used to train the algorithm, with a further five datasets used for testing, to ensure the quality of the trained AI for detecting features of naive data. The software was trained to use brightfield images to automatically detect the outline of zebrafish embryos, while concomitantly identify internal anatomical structures and regions (Fig. 1B). Currently, the internal anatomy detected includes eye, heart, tail fin, yolk sac, spine, bladder and otic vesicle (Fig. 1B). The embryo body is also subdivided into the head, trunk and tail regions. Each segmented region can be analysed for features such as morphology (i.e. eye size) or count (i.e. count one versus two eyes to determine orientation), and is combined with fluorescence quantification including intensity metrics and identification of labelled structures, such as fluorescent cells or granules (Fig. 1C).

This analysis approach also permits the automatic selection of wells containing, exclusively, the desired side-oriented fish (illustrated in Fig. 1D), without manual image inspection. Although the use of the alignment plate improves the number of fish in the correct orientation for analysis (Fig. 1A,B), a small number of fish in each plate are not properly aligned in the well [lying on their back, tail out of viewing window etc. (Fig. S2)]. This is usually <5% per plate. The side-orientated fish were selected using anatomical attributes identified by the Athena software, specifically those having both an eye count and a tail count equal to one, thereby excluding empty wells and fish in undesirable orientations. Defining a minimum fish and tail area in the software also excludes wells in which the fish is only partially in the well (see Materials and Methods). Automatic exclusion of these wells without any manual inspection of the images is a critical feature for high-throughput applications and avoids skewing statistics through incorrect or biased sample selection. While these improperly oriented fish could be correctly oriented via manual intervention, the required hands-on time far exceeds that needed to prepare, scan, process and analyse an additional 96-well plate and results in a lower total amount of useful data.

The rapid image acquisition combined with batch image processing and automatic detection of the fish and its anatomy allows for high-throughput analysis with minimal user input. The variety of features detected and the rapid, easy-to-use customisability of the application makes this platform a highly versatile tool for a number of screening applications, as we detail below.

### Accurate counts of HSPCs in the tail of 2-4 dpf zebrafish embryos

The primary goal of our screen was to accurately enumerate HSPCs, using the *Tg(itga2b:GFP)* reporter line (Fig. 1C). At 3 dpf, the HSPCs reside in the caudal haematopoietic tissue (CHT; analogous to fetal liver) in the base of the tail and in close proximity to the caudal-most tip of the yolk sac (Fig. 1D). We aimed to develop a screening platform that would provide an automatic count of the number of cells exclusively in this region to allow us to analyse the effects of myelodysplastic syndrome (MDS)-related genetic mutations that have been identified in the HSPCs of patients. Ultimately, such a tool will enable a large-scale small-molecule library screen to target mutant stem cells, to identify drugs that exclusively deplete the mutant HSPC population. One of the challenges in automating our assay is that zebrafish at 3 dpf have substantial auto-fluorescence, particularly around the head and yolk (Fig. 1C). The HSPCs of interest are the GFP^lo^ cells, whilst the GFP^hi^ cells are thrombocytes, some of which are in circulation (Lin et al., 2005). This meant that simply thresholding out the auto-fluorescence in the whole image erroneously eliminated a large number of cells of interest, or the inclusion of fluorescent spots not relevant to our question. The Athena Zebrafish Application can provide an automatic count of the fluorescent granules in a chosen region of the fish, in our case the tail (Fig. 1C, outlined in red), without manual inspection or segmentation of the images. Analysis of a 96-well plate takes ∼10 min. To verify the counts measured, we analysed a 96-well plate of wild-type transgenic zebrafish at 3 dpf with the Athena software and compared results to a manual cell count in the same images. The two counting methods were strongly correlated, r=0.844 (Fig. 1E), indicating that automated detection provides equivalent results to manual counting.

**Fig. 2.**
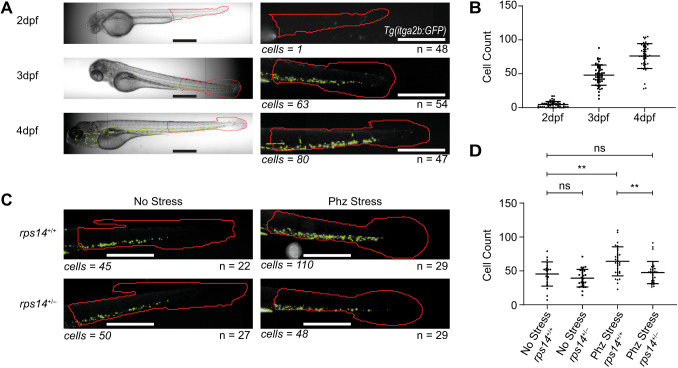
**Validation of HSPC counts using the Athena Zebrafish Application to detect differences in stem cell populations due to age or genetics of zebrafish embryos.** (A) Brightfield and fluorescent images of *Tg(itga2b:GFP)* embryos at 2-4 dpf and analysed for HSPC count (green) in the tail region (red). (B) HSPC counts at 2 dpf (*n*=48), 3 dpf (*n*=54) and 4 dpf (*n*=47), showing the increase in HSPC number over time. (C) Fluorescent images of the tail of *rps14^+/+^* and *rps14^+/−^ Tg(itga2b:GFP)* at 3 dpf following phz haemolytic stress for 24 h at 24 hpf alongside unstressed controls analysed for HSPC count (green) in the tail region (red). (D) HSPC count of the different conditions, showing no difference in HSPC count between unstressed *rps14^+/−^* (*n*=27) and unstressed *rps14^+/+^* (*n*=22) embryos, but an increase in HSPC count in the phz-stressed *rps14*^+/+^ embryos (*n*=29) compared with wild type, which does not occur in the phz-stressed *rps14*^+/−^ embryos (*n*=29), from triplicate experiments (Peña et al., 2020 preprint). Statistical analysis using unpaired *t*-tests. Error bars show mean±s.d. ns, *P*>0.05; **P<0.01. Scale bars: 500 µm. *n* refers to the number of embryos analysed for each condition. Cell counts give the Athena cell count for the example image shown.

Stem cells first migrate to the CHT at ∼2 dpf, where they then start to proliferate (Chen and Zon, 2009). We tested our platform on embryos of different ages to see the accumulation of HSPCs in the CHT over time (Fig. 2A). Using our automated HSPC counting assay, we confirmed that small numbers of HSPCs are present in the CHT at 2 dpf and this steadily increases over the next 48 h (Fig. 2A,B). In this experiment, different batches of embryos were used for each age. However, embryos are imaged alive and can easily be removed from the plate with a pipette, permitting longitudinal studies on the same embryo if desired. We also attempted to image embryos at 5 dpf; however, due to the inflation of the swim bladder, very few fish aligned in the desired side orientation, and our analysis could no longer be carried out with the desired throughput. The system could be used to image younger embryos, although this would require the use of a different plate, such as a round-bottomed plate, due to the shape of the embryo and the fragility of the yolk sack to centrifugation. Currently the software is trained to identify fish at 2-6 dpf, although with additional training datasets it could be trained to identify younger fish.

### Detection of phenotypic differences in HSPC and myeloid cell count

We have developed several models for MDS in zebrafish, including an Rps14 mutant line (Peña et al., 2020 preprint). In this model, heterozygous embryos are phenotypically indistinct from wild-type animals unless the animals are subjected to stress. Phenylhydrazine (phz) is used as a haemolytic stress in zebrafish, causing oxidation of haemoglobin and anaemia (Lenard et al., 2016; Shafizadeh et al., 2004; Ferri-Lagneau et al., 2012). If phz is applied for 24 h at 24-48 hours post-fertilisation (hpf), only the wild-type embryos recover from the induced anaemia (Schneider et al., 2016; Peña et al., 2020 preprint). Using our HSPC counting assay, we showed that, at 3 dpf, phz leads to an increase in HSPCs at 3 dpf in the wild types in response to anaemia, a response not observed in the Rps14 mutants [*P<*0.01, Fig. 2C,D (Peña et al., 2020 preprint)].

**Fig. 3.**
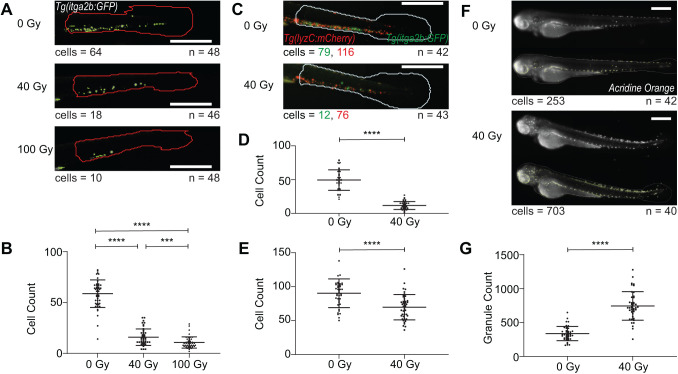
**Automated analysis of the effects of X-ray irradiation on HSPC count and myeloid count in dual fluorophore images, as well as apoptosis in zebrafish embryos.** (A) Fluorescent images of the tail of *Tg(itga2b:GFP)* embryos at 3 dpf, irradiated at 2 dpf with 0 Gy, 40 Gy or 100 Gy X-ray and analysed for HSPC count (green) in the tail region (red). (B) HSPC counts showing the reduction in HSPC number with 40 Gy (*n*=46) and 100 Gy (*n*=48) compared with 0 Gy (*n*=48). (C) Fluorescent images of the tail of *Tg(itga2b:GFP)(lyzC:mcherry)* dual fluorophore embryos at 3 dpf, irradiated at 2 dpf with 0 Gy or 40 Gy X-ray and analysed for HSPC (green) and myeloid (red) in the tail (white). (D,E) HSPC count of GFP-positive cells (D) and myeloid cell count of mCherry-positive cells (E) in the tail, showing the decrease in both cell types with irradiation (*n*=43) compared with unirradiated (*n*=42). (F) Fluorescent images of Acridine Orange-stained embryos at 3 dpf, irradiated at 2 dpf with 0 Gy or 40 Gy X-ray and analysed for granule count (green) in the whole fish. (G) Granule counts in the whole fish showing an increase in apoptosis following irradiation (*n*=40) compared with unirradiated (*n*=42). Irradiation experiments were performed in triplicate. Statistical analysis using unpaired *t*-tests. Error bars show mean±s.d. ***P<0.001, ****P<0.0001. Scale bars: 500 µm. *n* refers to the number of embryos analysed for each condition. Cell counts give the Athena cell count for the example image shown, for each cell type in dual fluorophore images.

To further define the sensitivity of the platform, we assessed the effects a range of non-lethal doses of X-ray irradiation on HSPC numbers in the embryo (Traver et al., 2004; McAleer et al., 2005). Embryos were irradiated at 2 dpf before imaging at 3 dpf. This led to a significant reduction in stem cells with 40 Gy and a further reduction with 100 Gy (Fig. 3A). The platform allows for swift analysis of these images in large numbers, and the phenotypic differences are easily measured with high statistical significance (*P*<0.001, Fig. 3B).

Beyond analysing solely HSPCs in our MDS models, we aimed to develop a versatile screening platform that we could easily extend to other assays including other zebrafish transgenics to label different cell types. We performed multiplexed fluorescence image acquisition using a double colour transgenic line with *Tg(itga2b:GFP)* and *Tg(lyzC:mCherry)*, which has mCherry-tagged myeloid cells (Buchan et al., 2019), for imaging in both the red and green channels (Fig. 3C). We used this fish line in the X-ray irradiation assay and identified both the GFP-tagged HSPCs and mCherry-tagged myeloid cells in the same fish (Fig. 3C) by imaging in both fluorescent channels and applying the fluorescent spot counting functionality to each of the two colours individually. Concomitant with the decrease in GFP-tagged HSPCs we also observed a decrease in mCherry-tagged myeloid cells after irradiation (Aldridge and Radford, 1998; Lehnert et al., 1985) (*P*<0.0001, Fig. 3D,E).

### Extended applications using fluorescent output – apoptosis, hair cell detection and angiogenesis

We wished to further assess the utility of our platform beyond haematological-related screens, to other assays that are commonly used for drug screening in zebrafish embryos, showing the broad applicability to a variety of screening applications. Apoptosis assays in zebrafish can be utilised for drug screens, including analysis of regulated and induced apoptosis and for assessing toxicity (McGrath and Seng, 2013; Parng et al., 2004). Acridine Orange is a fluorescent apoptosis marker that can easily be used to visualise cell death in zebrafish by incubation of live embryos in the stain followed by fluorescence imaging (Abrams et al., 1993; Furutani-Seiki et al., 1996; Tucker and Lardelli, 2007). Rapid automated quantification of this fluorescence could be a valuable tool for drug screening and toxicity assessment of chemical screens. To test whether our screening platform could be adapted to assess cell death, X-ray irradiation at 2 dpf was used to induce apoptosis in zebrafish embryos before analysis at 3 dpf. Irradiation in zebrafish embryos leads to apoptosis, with particular sensitivity towards cells within the spinal cord (Geiger et al., 2006). We labelled dying cells post-irradiation with the supravital stain Acridine Orange and imaged on the Hermes in high throughput (Fig. 3F). The Athena analysis pipeline was optimised by adjusting the threshold, smoothing and area parameters of the granule detection, to count the small stained granules that define the dying cell population in the whole fish. The increased cell death with irradiation was easily observed (*P*<0.0001, Fig. 3G).

**Fig. 4.**
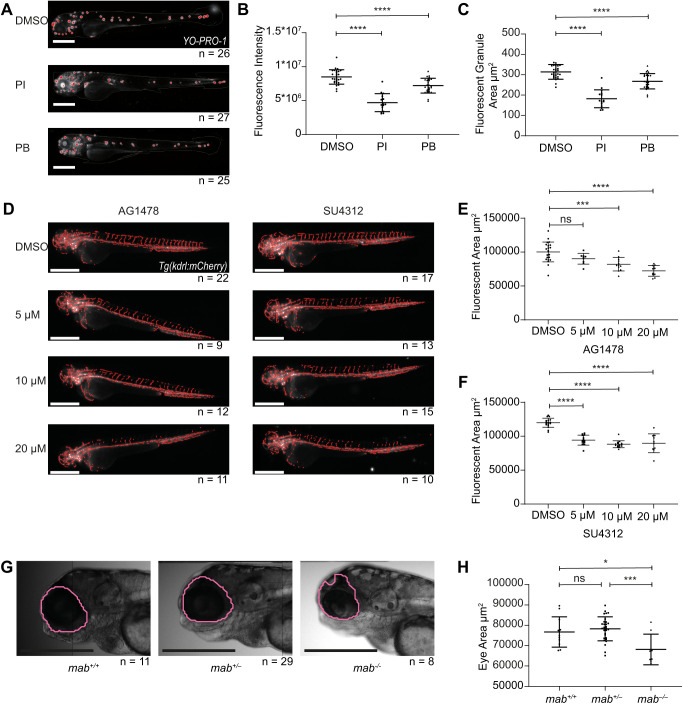
**Extended applications of the analysis platform including automated analysis of hair cell loss, angiogenesis and eye size in zebrafish embryos.** (A) Fluorescent images of YO-PRO-1-stained embryos at 4 dpf, following treatment for 1 h with DMSO, 100 µM pentamidine isethionate (PI) or 100 µM propantheline bromide (PB), analysed for fluorescent granules (red) in the whole fish. (B,C) Total fluorescence intensity in the whole fish (B) and average area of the fluorescent granules (C), showing decrease with drug treatment as previously described (Chiu et al., 2008). (D) Fluorescent images of *Tg(kdrl:mCherry)* embryos at 2 dpf, treated for 24 h from 24 hpf with DMSO, AG1478 or SU 4312 and analysed for mCherry fluorescence in the whole fish. (E,F) Total fluorescent area in the whole fish following treatment with AG1478 (E) and total fluorescent area in the whole fish following treatment with SU4312 (F), showing reduced angiogenesis in treated fish compared with the control, as previously described (Tran et al., 2007). (G) Brightfield images of *mab21l2^+/+^*, *mab21l2^+/−^* and *mab21l2^−/−^* mutant embryos at 4 dpf analysed for eye size (pink). (H) Eye area, showing the reduced eye size in mab21l2^−/−^ mutants. Results consistent with previous publications were achieved in a single experiment for each of these assays. Statistical analysis using unpaired *t*-tests. Error bars show mean±s.d. ns, *P*>0.05; *P<0.05, ***P<0.001, ****P<0.0001. Scale bars: 500 µm. *n* refers to the number of embryos analysed for each condition.

Assays analysing hair cells in the lateral line in zebrafish have also been utilised in drug discovery for compounds that mediate deafness and ototoxicity due to the similarities between these cells and the inner ear hair cells in humans (Whitfield, 2002; Nicolson, 2005; Ton and Parng, 2005). Hair cells in the lateral line are arranged into neuromasts along the head and body of the zebrafish and can be stained using the fluorescent marker YO-PRO-1. This has been utilised in several chemical and genetic screens (Chiu et al., 2008; Vlasits et al., 2012; Esterberg et al., 2013; Pei et al., 2018; Owens et al., 2008). To date, these screens have required manual evaluation of the neuromasts either by counting or scoring for degradation. We therefore sought to replicate the results of one of these screens using our automated analysis platform. Chiu et al. screened 1040 US Food and Drug Administration (FDA)-approved compounds for ototoxicity, and found that pentamidine isethionate (PI) and propantheline bromide (PB) reduced hair cell survival by almost 50% at 100 µM (Chiu et al., 2008). We incubated embryos at 4 dpf with 100 µM PI or PB for 1 h before staining with YO-PRO-1 and imaging on the Hermes. Both compounds led to a reduction in hair cells (Fig. 4A). Athena analysis of fluorescent granules contained within the whole fish was used to define regions of interest (the neuromasts) and measure their fluorescence intensity and area to examine the degradation of hair cells therein. Both compounds led to a statistically significant reduction in both integrated fluorescence intensity and fluorescent granule size (Fig. 4B,C, *P*<0.0001), in agreement with the results from Chiu et al. (2008).

Assays analysing angiogenesis, the formation of new blood vessels from pre-existing vessels, are also of therapeutic interest, with drug screening for antiangiogenic compounds of interest for the treatment of solid tumours (Teleanu et al., 2019; Zirlik and Duyster, 2018). Zebrafish provide a useful tool for *in vivo* drug screening for antiangeiogenic compounds (Serbedzija et al., 1999; Chávez et al., 2016), and transgenic zebrafish have previously been utilised to analyse the degree of angiogenesis in zebrafish embryos, identifying compounds that reduce blood vessel development (Tran et al., 2007; Mauro et al., 2019). We sought to assess whether our platform could be used to assess the degree of angiogenesis. Utilising *Tg(kdrl:mCherry)* fish, we treated embryos at 24 hpf for 24 h with either dimethyl sulfoxide (DMSO), AG1478 or SU4312, which were identified as antiangiogenic in zebrafish by Tran et al. (2007). At 48 hpf, the embryos were imaged on the Hermes, with both compounds leading to reduced angiogenesis (Fig. 4D). Athena was then utilised to identify areas of fluorescence, and the total area of the fluorescence within the whole fish used as a surrogate of the degree of vessel formation. As expected, both AG1478 and SU4312 led to a significant reduction in fluorescent area within the whole fish (Fig. 4E,F, *P*<0.0001), with the latter causing a more significant reduction at lower concentrations, consistent with the lower half-maximal inhibitory concentration (IC_50_) reported previously (Tran et al., 2007). A fibre identification algorithm could be introduced to Athena, to provide a more direct read out of angiogenesis.

### Brightfield phenotypic analysis – eye size

In addition to fluorescence assays, we assessed whether our platform could be adapted for morphological screens in brightfield. Mab21l2 is involved in healthy eye development, and zebrafish carrying homozygous *mab21l2* mutations display microphthalmia (smaller eyes) (Gath and Gross, 2019; Deml et al., 2015; Hartsock et al., 2014). Using an incross of *mab21l2^u517^* heterozygous embryos (Wycliffe et al., 2020), imaged in brightfield with the Hermes at 4 dpf, we used Athena to identify the eye in brightfield and measure the size. Consistent with previous studies, *mab21l2^u517^* homozygous eyes are smaller in size (Fig. 4G), as measured using Athena (*P*<0.05, Fig. 4H).

## DISCUSSION

Zebrafish embryos provide an excellent opportunity for *in vivo* screens, but often such screens rely on time-consuming manual assays or the development of bespoke automated solutions that require time and expertise to develop. Utilising the Hermes and Athena HCS platform from IDEA Bio-Medical offers an automated solution for zebrafish screens, which is both versatile and easy to use.

Image acquisition is straight forward to set up, with simple plate loading and imaging. The plates are loaded in the laboratory with a standard pipette, with no special treatment of the embryos required beyond the use of the alignment plate. Imaging is easy to carry out – the plate is placed in the microscope and the parameters chosen using user-friendly software, where changes can be visualised interactively before imaging. Imaging parameters can be saved and loaded to provide consistency between images acquired at any time. Unlike the VAST system, which offers 360° imaging of embryos in a capillary (Pulak, 2016; Pardo-Martin et al., 2010; Pardo-Martin et al., 2013; Chang et al., 2012), the Hermes microscope images the fish from beneath the plate. This allows for side-on multiplexed images of fish at 2-4 dpf (Fig. 2A) in multiple fluorescence colours (Fig. 3C). For many applications this is sufficient, and although not all embryos are perfectly aligned in the wells with our method, this is limited to ∼5% per plate, and the software parameters permit automatic exclusion of these wells without manual inspection of the images.

The Athena Zebrafish Application allows for easy, automated analysis of the acquired images. We have demonstrated that one of the principle advantages of this system is the ease with which customisation to different assays can be undertaken. Following setup with the HSPC assay, each of the subsequent optimisations shown in this manuscript were completed in a single experiment. We have shown that the system can accurately replicate published results from a number of different assays: HSPC counts in stressed and unstressed Rps14 mutant embryos (Peña et al., 2020 preprint) (Fig. 2C,D), hair cell degradation in drug-treated embryos (Chiu et al., 2008) (Fig. 4A-C), chemically induced reduction of angiogenesis (Tran et al., 2007) (Fig. 4D-F) and reduced eye size in *mab21l2* mutants (Wycliffe et al., 2020) (Fig. 4G,H). Acquisition and analysis protocols can be customised and then saved, allowing for consistency between experiments, an asset for high-throughput screens carried out over weeks or months.

Both image acquisition and analysis are extremely fast once the protocols have been saved. For a 96-well plate of embryos, image acquisition takes ∼15 min, image processing a further 20 min and image analysis around 10 min. The ability to screen and analyse large numbers of embryos in a single day with high consistency and such limited user input makes this platform particularly useful for high-throughput applications such as drug and genetic screens, where large numbers of embryos are required.

It is worth noting that a potential significant barrier for the use of this system is the cost, with both the microscope and the analysis packages being potentially out of reach of some laboratories. The flexibility across a large range of assays means the system can be both multi-purpose and multi-user, allowing it to be shared between groups. The Hermes microscope and Athena packages both have applications beyond zebrafish, with many applications in cell biology, which does gives potential for shared use across multiple laboratories and departments, and the opportunity to share the cost.

Zebrafish provide a unique opportunity for drug screening in whole live animals since they produce large numbers of fast-developing, transparent embryos. The development of an automated screening platform that is fast, customisable and easy to use without specialist knowledge or training will allow for much better utilisation of this unique system. As shown in this paper, the platform can be applied to a large number of different assays across a large range of research topics, with new assays being straightforward to develop using the user-friendly environment. The speed of acquisition and analysis allows access to larger compound libraries, the consistency between experiments allows for comparison of results across multiple experiments, and the ease of use makes the platform accessible to all. The training sets used to identify fish and anatomy are centralised and continuously updated/maintained, which also provides reproducibility and consistency between research groups.

This platform will allow for high-content screening in zebrafish, as well as being useful for more targeted experiments by allowing for the use of large numbers of embryos to give statistical power. The ease of use, speed of imaging and analysis, and consistency between images acquired at different times makes this platform useful for both small- and large-scale experiments. We now plan to use this platform for small-molecule drug screening, using the automated HSPC count presented here to conduct a synthetic lethal screen in an MDS-related mutant background.

## MATERIALS AND METHODS

### Zebrafish husbandry and experimental conditions

Zebrafish (*Danio rerio*) stocks were maintained according to standard procedures in UK Home Office-approved aquaria (Westerfield, 2007). Embryos were obtained from wild-type AB or AB/TL, transgenic strains *Tg(itga2b:GFP)*, which has green-fluorescent HSPCs (Lin et al., 2005), *Tg(lyzC:mcherry)*, which has red-fluorescent myeloid cells (Buchan et al., 2019), and *Tg(kdrl:mCherry)* (Choi et al., 2007), which has red-fluorescent vasculature, or mutant strains *rps14^E8fs^* (Peña et al., 2020 preprint) and *mab21l2^u517^* (Wycliffe et al., 2020). Embryos were staged according to Kimmel et al. (1995) and expressed in hpf/dpf. All procedures complied with UK Home Office guidelines.

### Preparation of embryos for imaging

At 24 hpf, embryos were dechorionated using pronase, and raised in 0.003% phenylthiourea-supplemented E_3_ medium to prevent pigment formation. At 2-4 dpf, embryos were anaesthetised with tricaine, and then loaded into a 96-well zebrafish alignment plate (Hashimoto ZF plate, Japan, 96-well) in 75 µl of the E_3_ medium using a wide orifice tip. The plates were then gently centrifuged at 200 ***g*** for 20 s before imaging.

### WiScan^®^ Hermes image acquisition

The 96-well plates were imaged using the WiScan^®^ Hermes High Content Imaging System (IDEA Bio-Medical, Rehovot, Israel). Images were taken in brightfield and green and red fluorescence channels at 4× magnification, with a well coverage of 150% and field density of 150%. This gave four overlapping images along each well. Brightfield was imaged with 35% light intensity, 40 ms exposure and 30% gain. Fluorescence channels were imaged with 90% light intensity, 200 ms exposure and 30% gain, except for *Tg(kdrl:mCherry)*, which was imaged with 50 ms exposure in the fluorescent channel. *Z*-stacks were taken in five planes with an inter-plane distance of 50.6 µm.

An accompanying image pre-processing software package (Advanced Data Processing software, IDEA Bio-Medical, Rehovot, Israel) was used to process the raw images of multiple datasets in batch prior to quantitative analysis. This software loads one or more image datasets obtained on the WiScan^®^ Hermes microscope, then performs one or more sequential image processing operations in batch. Image processing operations can include intensity projection (through time or *z*-slices), fluorescence deconvolution, sharpest (most in-focus) *z*-plane selection and image montage (stitching). Here, the sharpest *z*-slice for each raw field of view in the brightfield channel was selected, and maximum intensity projection for each raw field of view in the fluorescence channel was performed. Subsequently, all fields of view (sharpest brightfield and maximum fluorescence) in a well were stitched together to provide a single, two-colour channel image per well.

### WiSoft^®^ Athena image analysis

Image quantification was performed with the WiSoft^®^ Athena software Zebrafish Application (IDEA Bio-Medical, Rehovot, Israel). The software application performs automated, multiplexed image analysis by processing brightfield and fluorescence channels simultaneously, with different algorithms. Brightfield analysis of the whole zebrafish embryo utilised a novel deep learning-based AI algorithm to identify the fish and internal structures. The method is based on a supervised convolutional neural network and was trained using hundreds of images of 2-4 dpf zebrafish embryos as input, each paired with manually segmented ground-truth outputs. An option for custom, manual segmentation is also present in the Zebrafish Application. The resulting algorithm automatically identifies the fish outline, internal anatomical structures and three fish regions (head, trunk and tail). Users input only minimum and maximum size constraints to select the appropriate range for anatomical objects/regions that are to be identified and analysed. Fluorescence channels are analysed using image analysis techniques with optimised algorithms (smoothing, background subtraction, thresholding and area constraints; Table 1) to identify high signal-to-background objects, such as fluorescent granules.

**Table 1.**
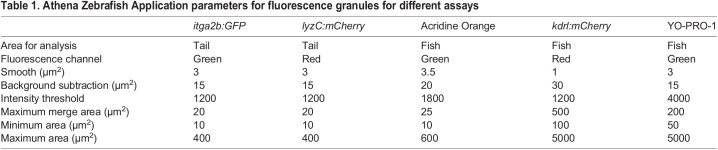
Athena Zebrafish Application parameters for fluorescence granules for different assays

The morphological features (length, area, shape, etc.) of the fish, organs and regions are quantified based on the structures identified in the brightfield channel. Objects (i.e. fluorescent granules) identified in the fluorescence channels that are contained within the fish outline are included in quantification, while spots outside the fish are disregarded. Fluorescent spots are quantified regarding their count and intensity within the fish as a whole, as well as within anatomical or regional structures (e.g. granule count within the tail, spot intensity within the spine, etc.).

Parameters were set to define the permitted size of detected fish and internal features in 2-4 dpf embryos (Table 2). No additional parameter input was required to identify these objects, since the AI algorithm was trained to identify them automatically. A population of on-side orientated fish for analysis was defined as those with an eye count and a tail count equal to one.

**Table 2.**
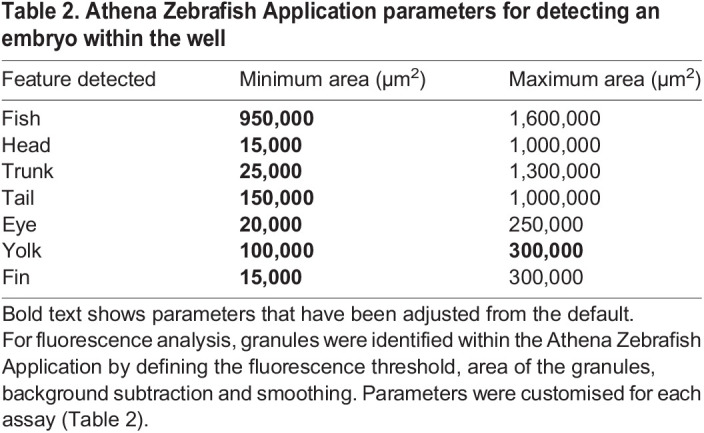
Athena Zebrafish Application parameters for detecting an embryo within the well

### Statistical analysis

For each experiment, the desired data were exported from Athena in csv format, for example granule in the tail count for HSPC count experiments, fluorescence area or eye size, for individual embryos. Statistical analysis was performed using GraphPad Prism v9.0.2. The probability level for statistical significance was *P*<0.05 and unpaired two-tailed *t*-tests were used for analysis. Data are presented as mean±s.d.

### Genotyping mutants

For *rps14* and *mab21l2* mutants, genotyping was required post-imaging. Embryos were removed in 50 µl of nuclease-free water using a multi-channel pipette and wide orifice tips and added directly to 1 µl 50× HotSHOT base solution (KOH 1.25 M, EDTA 10 mM). After incubation at 95°C for 30 min, the solution was neutralised with 1× HotSHOT neutralising solution (40 mM Tris-HCl). To identify the genotype of *rps14* and *mab21l2* mutants, we used the Kompetitive Allele Specific PCR (KASP) genotyping assay (LGC Genomics).

### X-ray irradiation

At 48 hpf, embryos were transferred to six-well plates for irradiation. X-rays (250 kV, 12.5 mA, 1.0 mm Al filter) for a total dose of 40 Gy or 100 Gy using an AGO HS 320/250 X-ray machine (AGO X-ray) equipped with an NDI-321 stationary anode X-ray tube (Varian). Embryos were placed back at 28°C for 24 h before imaging at 3 dpf as above.

### Acridine Orange staining for detection of apoptosis

At 3 dpf, live embryos in six-well plates were incubated in Acridine Orange (Invitrogen) staining solution (1 µg Acridine Orange in E_3_ medium) in the dark for 30 min with gentle rocking. Embryos were swiftly washed with E_3_ medium four times before being anaesthetised with tricaine, and then loaded into plates for imaging as above. Plates were kept in foil to shield from light and imaged promptly after staining.

### Hair cell assay

Protocol adapted from Chiu et al. (2008). At 4 dpf, live embryos in six-well plates were incubated in YO-PRO-1 (Invitrogen) staining solution (2 µM YO-PRO-1 in E_3_ medium) in the dark for 30 min with gentle rocking. Embryos were swiftly washed with E_3_ medium four times. Embryos were then treated with 100 µM PI, 100 µM PB or DMSO control for 1 h, anaesthetised with tricaine and loaded into plates for imaging as above.

### Angiogenesis inhibition assay

Protocol adapted from Tran et al. (2007). At 24 hpf, *Tg(kdrl:mCherry)* embryos were dechorionated with pronase and treated in six-well plates with AG1478 or SU4312 or DMSO for 24 h, then anaesthetised and loaded for imaging as above.

## Supplementary Material

Supplementary information
