# Supplementary figures and images for: A versatile, automated and high-throughput drug screening platform for zebrafish embryos
Source: Biol Open. 2021 Sep 2;10(9):bio058513. doi: 10.1242/bio.058513 (PMC8430230; doi:10.1242/bio.058513)

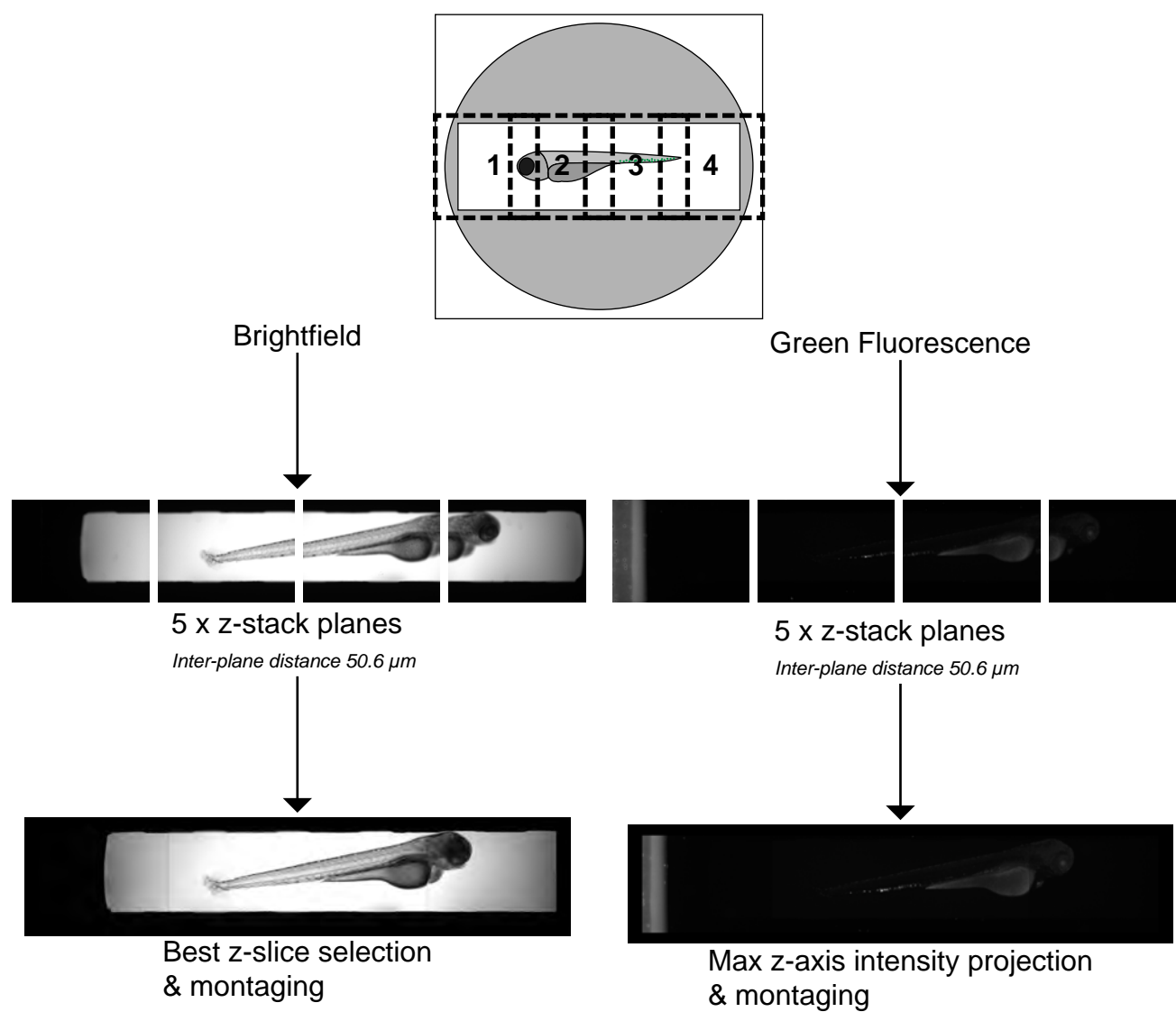

**Fig. S1**

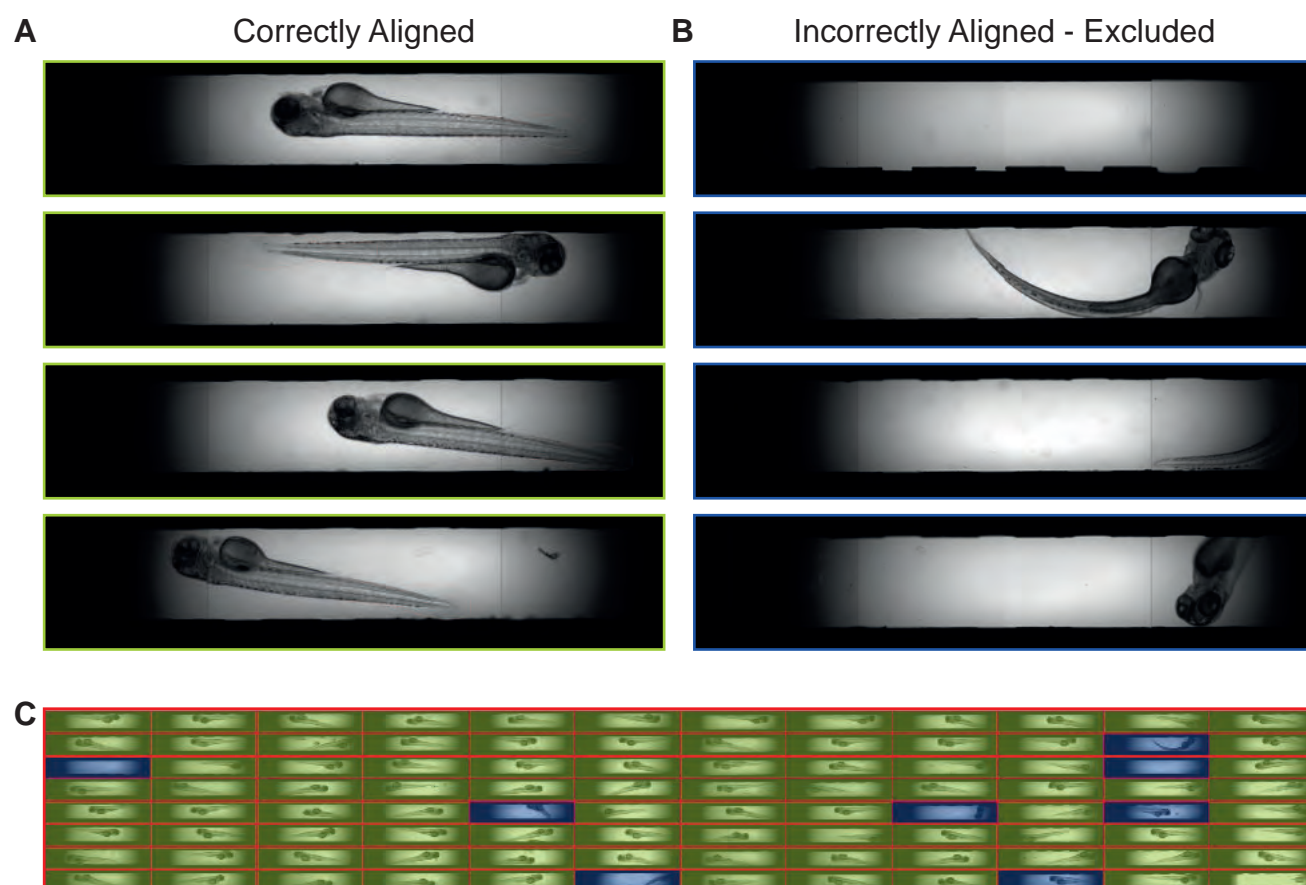

**Fig. S2.**

Supplement: Supplementary information [file biolopen-10-058513-s1.pdf]
